# Efficacy and safety of linagliptin in type 2 diabetes subjects at high risk for renal and cardiovascular disease: a pooled analysis of six phase III clinical trials

**DOI:** 10.1186/1475-2840-12-60

**Published:** 2013-04-09

**Authors:** Maximilian von Eynatten, Yan Gong, Angela Emser, Hans-Juergen Woerle

**Affiliations:** 1 Boehringer Ingelheim Pharmaceuticals, 900 Ridgebury Road, P.O. Box 368, Ridgefield, CT 06877, USA; 2 Boehringer Ingelheim Pharma GmbH & Co. KG, Binger Strasse 173, Ingelheim am Rhein, D-55216, Germany

**Keywords:** Type 2 diabetes mellitus, Hypertension, Microalbuminuria, Linagliptin, DPP-4 inhibitor

## Abstract

**Background:**

In patients with type 2 diabetes mellitus (T2DM), hypertension and microalbuminuria are predictive markers for increased renal and cardiovascular risk. This *post hoc* analysis of data from a global development program aimed to evaluate the efficacy and safety of linagliptin in a population with joint prevalence of these two vascular risk factors.

**Methods:**

Data for patients with baseline microalbuminuria (urine albumin-to-creatinine ratio 30–300 mg/g) and hypertension (systolic blood pressure ≥ 140 mm Hg and/or diastolic blood pressure ≥ 90 mm Hg and/or a history of hypertension; and/or an antihypertensive treatment at baseline) who participated in any of six randomized, placebo-controlled, phase III trials were analyzed. Participants received linagliptin 5 mg daily (alone or in combination with other oral antidiabetic drugs) or placebo for 18 to 24 weeks.

**Results:**

Of 3,119 patients, 512 had both microalbuminuria and hypertension (linagliptin, 366; placebo, 146). Baseline mean (SD) HbA1c was 8.3 (0.9)% and 8.4 (0.9)%; median (range) urine albumin-to-creatinine ratio was 60 (30–292) mg/g and 64 (30–298) mg/g; mean (SD) systolic blood pressure was 138 (15) mm Hg and 135 (16) mm Hg; and mean (SD) diastolic blood pressure was 81 (10) mm Hg and 81 (10) mm Hg, for linagliptin and placebo, respectively. Placebo-corrected mean change in HbA1c from baseline to week 18 and week 24 was -0.57% (95% CI: -0.75, -0.39; *P* < 0.0001) and -0.59% (95% CI: -0.80, -0.39; *P* < 0.0001), respectively. Placebo-corrected mean change in FPG from baseline to week 24 was -21.3 mg/dl (95% CI: -31.0, -11.6; *P* < 0.0001). The incidence of drug-related adverse events was similar for linagliptin and placebo (10.4% and 8.2%, respectively). Changes in systolic and diastolic blood pressure, cholesterol and triglyceride levels were similar between linagliptin and placebo.

**Conclusion:**

In T2DM patients with the two common vascular risk factors of hypertension and microalbuminuria, linagliptin achieved significant improvements in glycemic control. In this vulnerable patient population at high risk for micro- and macrovascular complications, linagliptin was well tolerated.

## Introduction

**Figure 1:**
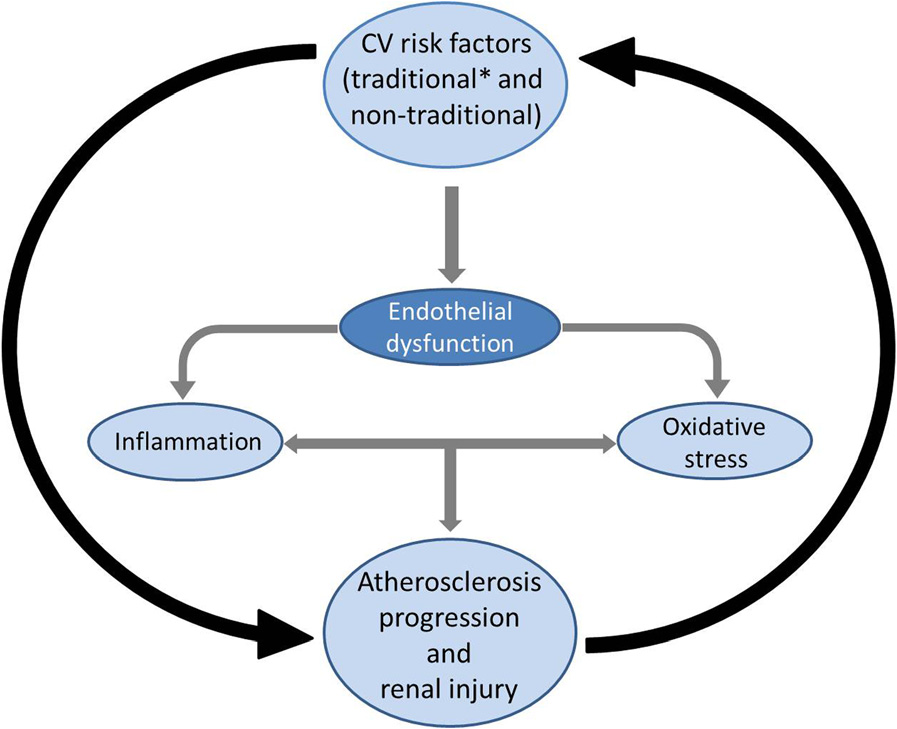
Cardiovascular risk factors and progression of renal dysfunction. *Diabetes, dyslipidemia, hypertension, obesity and metabolic syndrome. CV, cardiovascular Adapted from Chade AR, et al. *Hypertension *2005;45:1042–1049.

Type 2 diabetes mellitus (T2DM) affects over 300 million people worldwide and is the leading cause of renal and cardiovascular complications [1]. Despite lifestyle and pharmaceutical interventions, the management of hyperglycemia in patients at risk for renal and cardiovascular complications remains an important challenge to physicians treating this population [2]. In patients with T2DM and hypertension, urinary albumin is a key prognosticator of both renal and cardiovascular outcomes, with even low levels of albuminuria being associated with progressive renal dysfunction and increased risk of cardiovascular mortality [3]. Furthermore, traditional risk factors for atherosclerotic kidney disease, such as diabetes and hypertension, can lead to a cycle of declining renal function and progressive atherosclerosis (Figure 1) [4]. For these reasons, guidelines for the treatment of T2DM recommend testing for the presence of microalbuminuria – an early indicator of renal dysfunction – at initial diagnosis and in annual follow-up visits [5,6].

Linagliptin is an oral, once-daily dipeptidyl peptidase (DPP)-4 inhibitor that prevents the inactivation of incretin hormones glucagon-like peptide (GLP)-1 and glucose-dependent insulinotropic peptide, which stimulate glucose-dependent secretion of insulin. In large clinical trials undertaken in patients with T2DM, linagliptin as monotherapy or in combination with other oral antidiabetic drugs (OADs) has shown clinically meaningful efficacy with a low risk of hypoglycemia and no weight gain [7-9]. A meta-analysis of linagliptin phase III studies showed no increased cardiovascular risk with linagliptin [10]. A pooled analysis of data from eight phase III studies with linagliptin provided additional confirmation that linagliptin is well tolerated in patients with or without renal dysfunction [11]. Although renal dysfunction can increase risk for hypoglycemia, incidence of hypoglycemic events in patients receiving linagliptin without concomitant sulfonylurea was < 1.0% in this high-risk population [11].

Many OADs are primarily eliminated via the kidney, and require dosage adjustment based on renal function monitoring or become contraindicated when renal function declines further. Linagliptin has a primarily non-renal route of excretion. It can, therefore, be used without dosage adjustment irrespective of renal function [12], suggesting it is worthy of investigation in patients with, or at high risk of developing, renal as well as cardiovascular disease.

To further explore the effects of linagliptin in this high-risk population, data from patients with hypertension and microalbuminuria from six phase III clinical trials were pooled and analyzed.

## Methods

**Figure 2:**
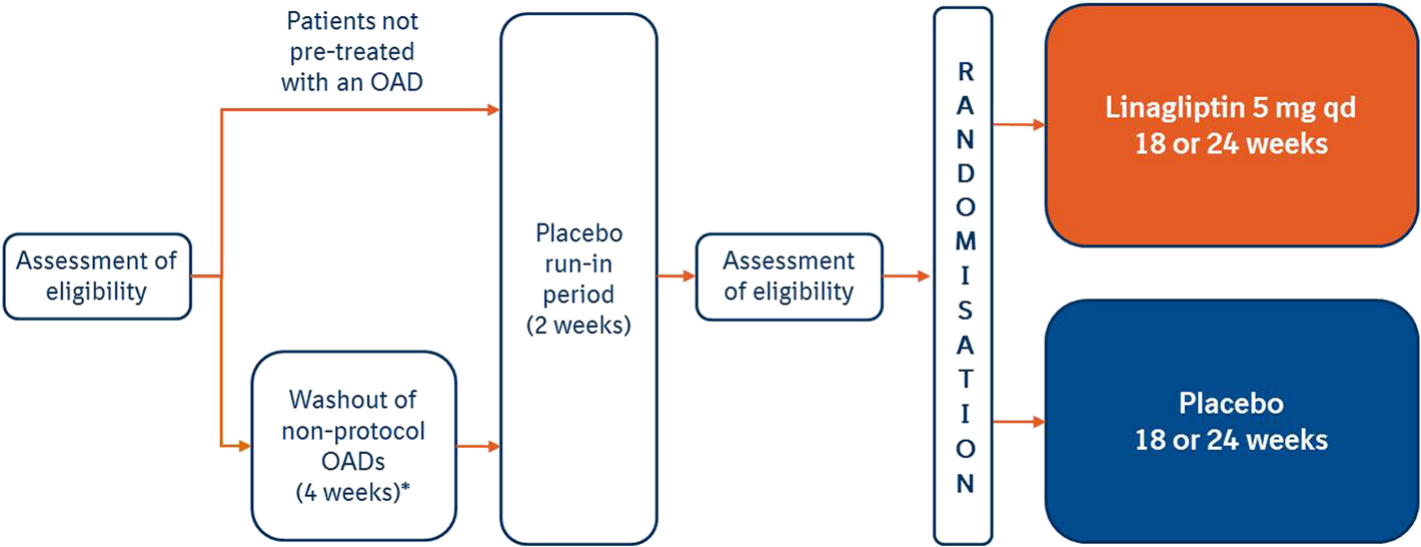
Schematic diagram of study designs. *No washout was performed in the studies adding linagliptin to existing treatment with metformin or sulfonylurea or both. Complementary study designs allowed pooling of the data.

This *post hoc* analysis pooled patient data from six randomized, double-blind, placebo-controlled, phase III trials from the linagliptin clinical trial program. These trials, ranging from 18 to 24 weeks (one 18-week trial included a 34-week active-controlled extension), were selected from those listed in the approved United States Food and Drug Administration prescribing information (indicated as an adjunct to diet and exercise to improve glycemic control in adults with T2DM) [12]. Patients with T2DM were treated with linagliptin 5 mg once daily or placebo as either monotherapy [7,13], add-on to metformin [9], add-on to sulfonylurea [14], add-on to metformin and a sulfonylurea [8], or as initial combination with pioglitazone [15]. All but one study comprised a 4-week washout period for those subjects taking non-protocol OADs, followed by a 2-week run-in period (Figure 2). In the study evaluating linagliptin added to metformin and a sulfonylurea, all patients directly entered the 2-week run-in period [8]. Following placebo run-in, patients were randomized to receive double-blind linagliptin 5 mg or placebo once daily.

All protocols were approved by relevant local independent ethical review or institutional review committees. Trials were carried out according to either the Declaration of Helsinki or International Conference on Harmonization Guideline for Good Clinical Practice. All patients provided written informed consent before participation.

Patients were included in this analysis if they had prevalent microalbuminuria (urine albumin-to-creatinine ratio [UACR] 30–300 mg/g, determined after randomization and prior to first drug intake by spot urine-quantitative measurement) and hypertension (systolic blood pressure [SBP] ≥ 140 mm Hg and/or diastolic blood pressure [DBP] ≥ 90 mm Hg and/or a history of hypertension; and/or antihypertensive treatment at baseline). The inclusion criteria for age and body mass index (BMI) were similar in the six trials: age of ≥ 18 and ≤ 80 years and BMI of ≤ 40 kg/m^2^. The inclusion criteria for glycated hemoglobin (HbA1c) levels at the start of placebo run-in ranged between ≥ 7% and ≤ 11%. In the majority of studies, OAD regimens, if any, needed to have remained constant for ≥ 10 weeks prior to enrolment [7-9,14].

Efficacy was assessed by the change from baseline in HbA1c and fasting plasma glucose (FPG) at 18 and 24 weeks of treatment. The analysis at week 18 was based on data from all six clinical trials, whereas the analysis at week 24 was based on data from four clinical trials [7-9,15].

This was also true for the UACR and blood pressure analyses of change from baseline. Lipid and blood pressure analyses were based on the last value on treatment (LVOT) data. Safety endpoints included the frequency and intensity of adverse events (AEs) and the frequency of hypoglycemic events.

### Statistical analysis

Efficacy endpoints were evaluated with an analysis of covariance (ANCOVA). The general model contained the following factors: treatment, washout of prior OAD and study, as well as the linear covariate continuous baseline HbA1c. For the FPG analysis, continuous baseline FPG was also included in the general model. The efficacy endpoints were assessed on the full analysis set (FAS), comprising all randomized subjects who received ≥ 1 dose of study drug and had both baseline and ≥ 1 on-treatment HbA1c measurement. A last observation carried forward (LOCF) approach was used to handle missing values for the calculation of the placebo-corrected mean change in HbA1c at week 18 and week 24 and the placebo-corrected mean change in FPG at week 24. The placebo-corrected mean change in FPG at week 18 was presented as observed cases (OC) because LOCF data were not available from all included trials for that time point. Efficacy measurements after start of rescue were replaced by missing values. Pooled safety data were recorded from the treated set (all randomized subjects who received ≥ 1 dose of study drug) and were analyzed using descriptive statistics.

## Results

### Patient demographics

Across the six studies, 3,119 patients were treated with either linagliptin 5 mg once daily (2,222) or placebo (897). Among that population, 512 patients were identified as having both microalbuminuria and hypertension at baseline and were therefore eligible for inclusion in the pooled analysis. At baseline, patient demographics and clinical characteristic were similar in the linagliptin and placebo groups (Table 1). In the overall evaluated population patients had a mean age of 59.5 years, with mean BMI of 29.9 kg/m^2^. Baseline mean (SD) HbA1c and FPG were similar in both treatment groups (HbA1c: linagliptin, 8.3 [0.9]% and placebo, 8.4 [0.9]%; FPG: linagliptin, 176 [52] mg/dl and placebo, 178 [39] mg/dl). At baseline, the majority of patients were being treated with two OADs (48.6%) and had known diabetes for > 5 years (57.6%). The median (range) UACR at baseline was 60 (30–292) mg/g for linagliptin and 64 (30–298) mg/g for placebo. The mean (SD) SBP was 138 (15) mm Hg and 135 (16) mm Hg, and mean (SD) DBP was 81 (10) mm Hg and 81 (10) mm Hg for linagliptin and placebo, respectively.

**Table 1:** Baseline patient demographics and clinical characteristics (treated set)

**Values are mean (±SD) or % of subjects**	**Linagliptin 5 mg (*n*** **= 366)**	**Placebo (*n*** **= 146)**
Age, years	59.8 (10.2)	58.8 (9.8)
Male, %	50.0	58.2
Race, %		
American Indian/Alaska Native	0.5	0.7
Asian	36.3	34.9
Black/African American	1.6	1.4
White	61.5	63.0
HbA1c, %	8.3 (0.9)	8.4 (0.9)
FPG, mg/dl*	176 (52)	178 (39)
BMI, kg/m^2^	29.9 (5.1)	29.9 (4.9)
eGFR, %		
≥ 90 ml/min	50.5	54.8
60 to < 90 ml/min	41.5	38.4
30 to < 60 ml/min	7.9	6.8
Urine albumin-to-creatinine ratio, mg/g, median (range)^†^	60 (30–292)	64 (30–298)
SBP, mm Hg	138 (15)	135 (16)
DBP, mm Hg	81 (10)	81 (10)
Metabolic syndrome, %	64.8	60.3
Time since diagnosis of diabetes, %		
Up to 1 year	11.2	13.7
> 1 to 5 years	29.5	32.9
> 5 years	59.3	53.4
Number of prior antidiabetes drugs, %		
0	13.7	24.0
1	35.5	31.5
2	50.3	44.5
3	0.5	0.0
Antihypertensive therapy, %	86.3	84.9
Beta-blockers	29.8	23.3
ACE inhibitors	40.4	41.1
ARBs	21.0	19.9
Diuretics	19.1	21.2
Calcium antagonists	27.6	27.4
Combinations	13.4	13.7

*Linagliptin, *n* = 358; placebo, *n* = 144.

^†^Linagliptin, *n* = 348; placebo, *n* = 138.

eGFR, estimated glomerular filtration rate, according to the Modification of Diet in Renal Disease study equation; ACE, angiotensin-converting enzyme; ARB, angiotensin-II receptor blocker.

Antihypertensive therapy was taken by 86.3% and 84.9% of patients in the linagliptin and placebo groups, respectively. The most commonly reported antihypertensive drugs were angiotensin-converting enzyme (ACE) inhibitors (linagliptin, 40.4% and placebo, 41.1%).

### Efficacy

**Figure 3:**
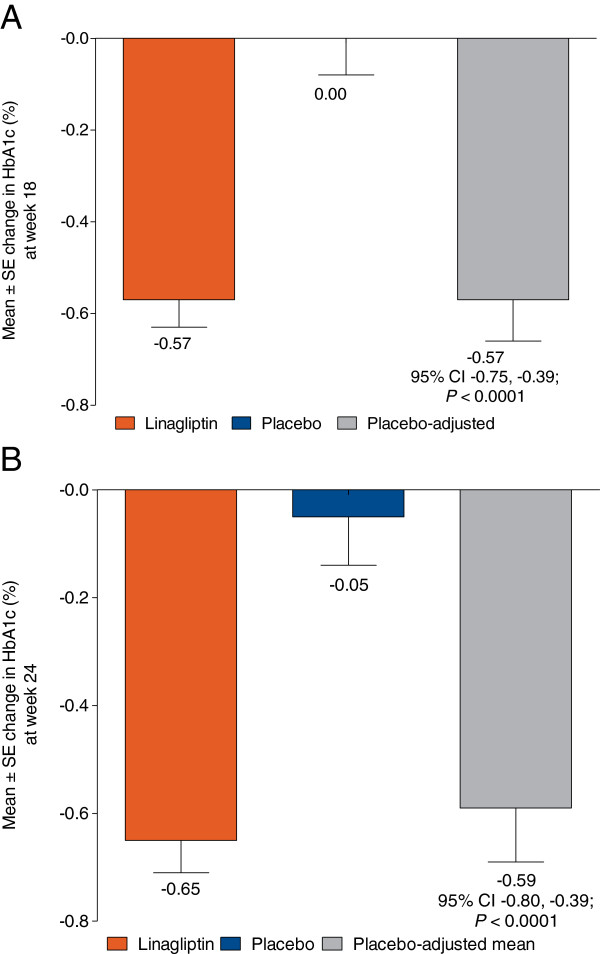
Change from baseline in HbA1c at week 18 (A)^* ^**and week 24 (B)**^**† **^(FAS LOCF). *Data based on all six clinical trials. ^†^Data based on four clinical trials.

Among patients with microalbuminuria and hypertension, those treated with linagliptin achieved a significantly greater reduction in HbA1c from baseline compared with placebo (Figure 3). The adjusted mean change from baseline (SE) in HbA1c at week 18 was -0.57 (0.06)% for linagliptin and 0.0 (0.08)% for placebo, resulting in a placebo-corrected mean change from baseline in HbA1c of -0.57% (95% confidence interval [CI]: -0.75, -0.39; *P* < 0.0001). At week 24, the adjusted mean change from baseline in HbA1c was -0.65 (0.06)% and -0.05 (0.09)% in the linagliptin and placebo groups, respectively. In these patients, the placebo-corrected mean change from baseline in HbA1c was -0.59% (95% CI: -0.80, -0.39; *P* < 0.0001) at week 24.

Linagliptin was also better than placebo in lowering FPG levels in this population. Adjusted mean change from baseline (SE) in FPG at week 18 was -12.3 (2.3) mg/dl for linagliptin and -1.6 (3.5) mg/dl for placebo, resulting in a placebo-corrected mean change from baseline of -10.6 mg/dl (95% CI: -18.4, -2.9; *P =* 0.0074). At week 24, the adjusted mean change from baseline in FPG was -13.4 (2.9) mg/dl and 7.9 (4.4) mg/dl in the linagliptin and placebo groups, respectively. The placebo-corrected mean change from baseline in these patients was -21.3 mg/dl (95% CI: -31.0, -11.6; *P* < 0.0001).

### Safety and tolerability

In the treated set, the overall incidences of any AE and serious AEs were comparable between both treatment groups (linagliptin, 62.6% and placebo, 62.3%; linagliptin, 4.1% and placebo, 6.2%). Investigator-determined drug-related AEs were reported by 10.4% and 8.2% of the linagliptin and placebo groups, respectively.

**Table 2:** Adjusted mean change* from baseline to last value on treatment in total cholesterol, LDL cholesterol, HDL cholesterol, and triglycerides

		**Linagliptin 5 mg (*n*** **= 366**^**†**^**)**	**Placebo (*n*** **= 146**^**†**^**)**
		**Mean (SE)**	**Mean (SE)**
**Total cholesterol, mg/dl**	**Baseline**	180.4 (1.1)	177.2 (1.6)
	**Change from baseline**	-0.4 (1.0)	2.1 (1.3)
	**Placebo-corrected difference**	-1.8 (1.4)	—
	**95% CI**	-4.4, 0.9	—
	***P *value**	0.195	—
**LDL, mg/dl**	**Baseline**	144.5 (1.9)	142.3 (2.9)
	**Change from baseline**	2.2 (1.8)	4.1 (2.3)
	**Placebo-corrected difference**	-1.8 (2.4)	—
	**95% CI**	-6.6, 3.0	—
	***P *value**	0.452	—
**HDL, mg/dl**	**Baseline**	40.2 (1.1)	40.0 (1.9)
	**Change from baseline**	1.5 (0.6)	2.6 (0.8)
	**Placebo-corrected difference**	-1.2 (0.8)	—
	**95% CI**	-2.8, 0.5	—
	***P *value**	0.172	—
**Triglycerides, mg/dl**	**Baseline**	272.6 (12.5)	241.0 (10.1)
	**Change from baseline**	-18.5 (7.7)	-13.5 (9.9)
	**Placebo-corrected difference**	-5.0 (10.6)	—
	**95% CI**	-25.9, 15.9	—
	***P *value**	0.6360	—
**SBP, mm Hg**	**Baseline**	137.8 (0.8)	134.8 (1.3)
	**Change from baseline**	-2.9 (0.9)	-2.4 (1.2)
	**Placebo-corrected difference**	-0.6 (1.3)	—
	**95% CI**	-3.1, 2.0	—
	***P *value**	0.664	—
**DBP, mm Hg**	**Baseline**	80.5 (0.5)	81.4 (0.8)
	**Change from baseline**	-1.2 (0.6)	-1.0 (0.8)
	**Placebo-corrected difference**	-0.2 (0.8)	—
	**95% CI**	-1.9, 1.4	—
	***P *value**	0.773	—

*Adjusted for baseline HbA1c, parameter measured, prior OADs, study and treatment.

^†^Patient numbers varied for each parameter. For linagliptin, total cholesterol n = 351, LDL n = 349, HDL n = 348, triglycerides n = 349, SBP n = 359, DBP n = 359. For placebo, total cholesterol n = 140, LDL n = 139, HDL n = 140, triglycerides n = 139, SBP n = 144, DBP n = 144.

DBP, diastolic blood pressure; HDL, high-density lipoprotein; LDL, low-density lipoprotein; LVOT, last value on treatment; SBP, systolic blood pressure.

AEs leading to discontinuation of trial medication were reported by 1.4% and 3.4% of patients in the linagliptin and placebo groups, respectively. Less than 1% of subjects experienced hypoglycemia when linagliptin was administered as monotherapy or in addition to metformin or pioglitazone. However, when linagliptin was administered with a sulfonylurea, the number of patients experiencing hypoglycemia was greater than with placebo (linagliptin, 19.8% and placebo, 5.9%).

The median (range) change in UACR from baseline at week 24 was -13.7 (-240.4 to 695.7) mg/g for linagliptin and -4.9 (-234.3 to 2,263.9) mg/g for placebo. The placebo-corrected median change from baseline in UACR at week 24 was -8.8 mg/g.

Mean (SD) SBP decreased by -2.9 (0.9) mm Hg and -2.4 (1.2) mm Hg in the linagliptin and placebo groups, respectively, at LVOT (Table 2). Mean (SD) DBP decreased by -1.2 (0.6) mm Hg and -1.0 (0.8) mm Hg in the linagliptin and placebo groups, respectively, at LVOT.

Minor changes were observed in lipid parameters in the linagliptin group as compared to the placebo group (Table 2). Total cholesterol, low-density lipoprotein and high-density lipoprotein showed small but non-significant differences between the groups. Triglyceride levels decreased in both treatment groups, with a numerically greater decrease seen with linagliptin (-18.5 mg/dl vs. -13.5 mg/dl).

No deaths occurred in either treatment group of the population of patients with microalbuminuria and hypertension. The incidence of the composite endpoint of adjudicated cardiovascular death, myocardial infarction and stroke was 0.27% (*n* = 1) and 0.68% (*n* = 1) in the linagliptin and placebo groups, respectively. A list of AEs by system-organ class is presented in Table 3.

**Table 3:** Summary of clinical AEs and hypoglycemia (treated set)

**% Subjects**	**Linagliptin 5 mg (*n*** **= 366)**	**Placebo (*n*** **= 146)**
***Clinical AEs***		
Any AE	62.6	62.3
Investigator-defined drug-related AE	10.4	8.2
Any AEs classified by system organ class*		
Gastrointestinal disorders	11.5	15.8
Infections and infestations	21.6	22.6
Nasopharyngitis	5.7	4.8
Injury, poisoning and procedural complications	7.4	8.2
Metabolism and nutrition disorders	21.0	20.5
Musculoskeletal and connective tissue disorders	11.2	11.0
Nervous system disorders	8.2	12.3
Respiratory, thoracic and mediastinal disorders	4.6	5.5
Vascular disorders^†^	7.1	4.8
AEs leading to study drug discontinuation	1.4	3.4
Serious AEs	4.1	6.2
Deaths	0.0	0.0
***Hypoglycemia***		
Subjects with hypoglycemia	9.3	2.1
Subjects with hypoglycemia by study		
Studies without sulfonylurea^‡^	0.5	0.0
Studies with sulfonylurea^§^	19.8	5.9
Severe hypoglycemia^¶^	0.3	0.0

*Medical Dictionary for Regulatory Activities, version 14.0.

^†^Individual AEs (preferred terms; %) were accelerated hypertension (linagliptin 0.0, placebo 0.7), arteriosclerosis (linagliptin 0.5, placebo 0.0), hypertension (linagliptin 5.2, placebo 4.1), hypertensive crisis (linagliptin 1.1, placebo 0.0), temporal arteritis (linagliptin 0.3, placebo 0.0). 2 of these events were considered drug-related (2 cases of hypertension; 1 with linagliptin and 1 with placebo).

^‡^Linagliptin, *n* = 199; placebo, *n* = 95.

^§^Linagliptin, *n* = 167; placebo, *n* = 51.

^¶^Event requiring assistance of another person to actively administer carbohydrate, glucagon or other resuscitation.

## Discussion

Vascular complications are the main challenge in the management of T2DM. In patients with T2DM, hypertension and prevalent microalbuminuria are common clinical features that guide treating physicians in assessing risk of cardiovascular and renal outcomes. Clinical evidence has shown a clear transitional path from microalbuminuria to chronic kidney disease [16] and a continuous relationship between albuminuria of any degree and increased cardiovascular mortality [17,18].

In the United Kingdom Prospective Diabetes Study, one quarter of patients developed microalbuminuria within 10 years of being diagnosed with T2DM [16]. Manifestations of urinary albumin progressed from normoalbuminuria to microalbuminuria at an annual rate of 2.0%, from microalbuminuria to macroalbuminuria at 2.8% annually, and from macroalbuminuria to diabetic nephropathy at 2.3% annually. Among patients with microalbuminuria, the study found an annual cardiovascular mortality rate of 2.0% compared with 3.5% in those with macroalbuminuria.

International guidelines for the treatment of T2DM recommend reducing the risk or slowing the progression of kidney disease through optimization of glycemic control [19]. A variety of pharmaceutical options for reduction of hyperglycemia are available, including metformin, sulfonylureas and incretin-based therapies (GLP-1 analogs and DPP-4 inhibitors). For patients with T2DM, however, renal impairment can be a limiting factor in the selection of appropriate antihyperglycemic therapies. Metformin and some sulfonylureas have contraindications or recommended dose adjustments related to renal impairment. Dose adjustment is also recommended for all DPP-4 inhibitors except linagliptin when used in patients with moderate to severe renal impairment [12,20]. Linagliptin, due to its predominantly non-renal route of elimination, requires no dose adjustment.

In this *post hoc* analysis of 512 patients with T2DM at high renal and vascular risk (defined as prevalent hypertension and microalbuminuria with T2DM), linagliptin showed significant and clinically relevant reductions in HbA1c and FPG in comparison with placebo. These findings were comparable to the efficacy results found in the individual six phase III trials [7-9,13-15].

Along with these efficacy results, this analysis showed that linagliptin was well tolerated. At baseline, patients in the two treatment groups had well-matched clinical characteristics and similar backgrounds of antihypertensive therapies, with approximately 85% of each group receiving at least one drug in that class and 13% of each group receiving combination antihypertensive therapy. The incidence of hypoglycemia was markedly greater in the linagliptin group only when linagliptin was administered with sulfonylurea, which is consistent with clinical findings for other DPP-4 inhibitors [8,21,22]. This tendency may be attributable to the proposed uncoupling effect that sulfonylureas have on the glucose-dependent effects of incretin therapies [23]. The percentage of patients receiving linagliptin who experienced serious AEs or AEs related to study drug was similar to those receiving placebo. Gastrointestinal AEs and infections, which are of concern in the DPP-4 class, occurred in similar proportions of patients in the linagliptin and placebo groups. No deaths occurred in either group.

Research suggests that GLP-1 may have beneficial effects on dyslipidemia, and recent small studies with DPP-4 inhibitors have shown favorable effects [24,25] or a neutral effect [26] on postprandial dyslipidemia in patients with T2DM. The present analysis showed a reduction in mean triglyceride levels with linagliptin at LVOT, which was numerically greater than that seen with placebo.

Linagliptin had a modest effect on blood pressure, comparable to studies with other DPP-4 inhibitors, which have shown small to neutral effects on blood pressure [24,26-28]. Minor changes were also observed in UACR with linagliptin treatment. In a recent *post-hoc* analysis of pooled Phase III data, linagliptin significantly reduced UACR after 24 weeks compared with placebo [29]. The change in mean UACR versus baseline was -32% (95% CI: -42, -21; *P* < 0.05) with linagliptin. Patients in this earlier analysis were defined as having albuminuria ranging from micro- (UACR, >30 to ≤300 mg/g) to macroalbuminuria (UACR >300 to ≤3000 mg/g) and were on stable ACE inhibitor/angiotensin II receptor blocker background therapy. Hence, differences in baseline UACR levels along with more frequent concomitant treatment with renin–angiotensin–aldosterone system inhibitors may have contributed to the larger reduction in UACR in that analysis. In order to specifically evaluate the albumin-lowering potential of linagliptin a randomized, controlled trial has recently been initiated (clinicaltrials.gov: NCT01792518, MARLINA). So far, however, research on the use of DPP-4 inhibitors in patients with renal dysfunction or albuminuria has been limited. The present analysis did not evaluate the efficacy or safety of linagliptin by subgroups of renal function, but baseline eGFR data were included to provide a full description of the clinical characteristics of the population with both microalbuminuria and hypertension. DPP-4 inhibitors have previously been shown to be efficacious and well tolerated at reduced doses in patients with moderate to severe renal impairment [30,31]. In clinical trial populations that included patients with mild and moderate renal impairment, linagliptin has been shown to be an efficacious and well-tolerated treatment without dose adjustment [7,9,15]. A phase I clinical trial with linagliptin showed that renal impairment ranging from mild to end-stage renal disease had no clinically meaningful effects on its pharmacokinetics [32]. Additionally, a recently published phase III trial of patients with severe renal impairment, demonstrated that treatment with linagliptin 5 mg daily provided clinically meaningful reductions in HbA1c over 1 year (-0.7%, 95% CI: -21.0 to -20.4; *P* < 0.0001) with similar tolerability to placebo [33].

In patients treated with linagliptin in the present analysis, incidence of severe hypoglycemia was very low, body weight and renal function remained stable, and no cases of drug-related renal failure were reported, suggesting that linagliptin can be used safely in all patients and even in this highly susceptible patient population with severe renal impairment.

The findings of this study are limited by the *post hoc* pooled nature of the analysis. Patients who participated in any of six randomized, placebo-controlled, phase III trials, had different background antidiabetic therapies. The analysis was also based on a relatively short duration of 24 weeks or less, which does not allow for long-term safety assessments. However, taken together with previous reports of up to 2 years in patients with T2DM [7-9,13-15,34], these results provide further evidence of beneficial glycemic effects and tolerability in this population. The ongoing CAROLINA trial (clinicaltrials.gov: NCT01243424) is investigating long-term cardiovascular outcomes with linagliptin. This trial will include patients with vascular-related end-organ damage (such as moderate renal dysfunction or microalbuminuria) as well as patients with prevalent cardiovascular risk factors, such as hypercholesterolemia or hypertension, and will allow for long-term safety assessments. It is the first long-term, clinical evaluation of a DPP-4 with an active comparator.

In conclusion, in patients with T2DM complicated by hypertension and microalbuminuria, linagliptin 5 mg once daily is well tolerated and improves glycemic control. Linagliptin may support long-term metabolic therapeutic strategies to treat patients at high risk of renal and cardiovascular disease.

## Competing interest

All of the authors are employees of Boehringer Ingelheim Pharmaceuticals.

## Authors’ contribution

MvE, YG, AE and HJW participated in the analysis of data and the review and editing of this manuscript. All authors read and approved the final manuscript.
